# Early and advanced stages of Maillard reaction in infant formulas: Analysis of available lysine and carboxymethyl-lysine

**DOI:** 10.1371/journal.pone.0220138

**Published:** 2019-07-24

**Authors:** Kataneh Aalaei, Ingegerd Sjöholm, Marilyn Rayner, Cristina Teixeira, Eden Tareke

**Affiliations:** 1 Department of Food Technology, Engineering and Nutrition, Lund University, Lund, Sweden; 2 Food for Health Science Centre, Lund University, Medicon Village, Lund, Sweden; Fisheries and Oceans Canada, CANADA

## Abstract

Although the literature on the Maillard reaction in infant formulas is extensive, most studies have focused on model systems, and in only a few cases on real food systems. Therefore, the objective of the present study was to determine the status of the Maillard reaction, both the early and advanced phases, in a variety of commercial infant formulas available on the Swedish market. Ten powder and liquid milk-based infant formulas from three manufacturers were selected to determine available lysine and CML contents, the two established indicators of the reaction. The products were also characterized with respect to protein content, carbohydrates composition, water content and water activity. In order to be able to compare the impact of different processing steps applied on powder and liquid formulas, the solid formulas contained similar ingredients as their corresponding liquid ones. Our findings showed that powder and liquid formulas contained similar available lysine concentrations regardless of the manufacturer, showing 27.14–36.57% decrease in the available lysine, compared to the reference skim milk powder in this study. The CML concentrations were in a broad range of 68.77–507.99 mg / kg protein. In the case of one manufacturer, liquid infant formulas had significantly higher CML content, compared to the powder products (p < 0.05). The results from this study are a step taken towards better understanding of the extent of the Maillard reaction in real complex systems of infant formulas.

## Introduction

Infant formulas (IF) developed as substitutes for human milk are usually based on cow’s milk and cow’s milk-derived ingredients. They are mainly composed of milk proteins (casein and whey proteins), lactose, vegetable oils, starch, and cereal proteins such as wheat, oat, maize as well as minerals and vitamins to meet the nutritional requirements of infants. The difference between cows’ and humans’ milk is in the higher lactose content of the latter (7.1%), compared to the corresponding value for cow’s milk (4.6%). Furthermore, the composition of human milk protein is 40% casein and 60% whey proteins, compared to cow’s milk protein which contains 80% casein and 20% whey proteins [1]. These differences are the main criteria taken into consideration for the formulation of IFs.

To ensure bacteriological safety of the IFs, these products are exposed to a wide variety of heat treatments including pasteurization, in bottle sterilization (110°C, 10 min), sterilization through ultra-high temperature processing (UHT) (130–140°C, 3–6 s), spray-drying (inlet temperature of 170–200°C) [2], and any combination there of. Therefore, due to their composition and exposure to the rigorous thermal processing, these products are more prone to the chemical reactions, among which non-enzymatic browning or the Maillard reaction has captured considerable attention [3]. This reaction involves reducing sugars and amino acids in a complex network of reaction pathways which leads to the formation of dietary advanced glycation end products (AGEs) [4]. AGEs are a group of molecules that when binding with the receptor for advanced glycation end products (RAGE), the main receptor of AGEs in the body, potentially activate a pro-inflammatory status, which in long term may lead to the development of the chronic inflammatory diseases such as diabetes, renal disease, and coronary heart disease [5].

There are studies showing that processing of the IFs, especially the application of sterilization and drying induce the Maillard or the so called non-enzymatic glycosylation reaction in these products [1]. To understand the occurrence and progression of these reactions, different markers have been the focus of several studies. Lysine, the essential amino acid is one of the main reactants which become unavailable or blocked in the beginning of the reaction via interaction with lactose. Histidine with its imidazole group, and arginine with a guanidine group are the other reactants [6]. Therefore available lysine, the established marker of early stage of the Maillard reaction has been utilized in several studies [7–13].

There are also studies in the literature focusing on the advanced stage of the Maillard reaction and formation of AGEs in IFs through analysis of the related indicators such as carboxymethyl-lysine (CML) and pyrraline [14–17]. However, research on both the early and advanced stages of the Maillard reaction in real complex systems of infant formulas are scarce.

The objective of the present study was thus, to determine the status of the Maillard reaction, both the early and advanced phases, in selected commercial infant formulas available on the market. Thereby, ten powder and liquid milk-based infant formulas from three manufacturers on the Swedish market were chosen and were analyzed using our previously validated procedures [6, 18] to determine both the available lysine and CML contents. The aim was to compare powder and liquid infant formulas, and to improve our understanding of the impact of different processing steps applied on powder and liquid IFs on the decrease of available lysine and formation of CML as a well-characterized AGE molecule.

## Materials and methods

### Samples

The study was carried out using four powder and six liquid infant formulas from three manufacturers A, B and C (Table 1). Powder samples 1–4 contained similar ingredients as liquid samples 5–8 respectively which made it possible to compare the impact of processing on the progression of the Maillard reaction in these products. The common ingredients were skim milk powder, whey powder, vegetable oil, starch, maltodextrin, vitamins, minerals and either oat or maize were the other protein source. All the products had been in the first third of their best before time period at the time of purchase, and had significant time (6–9 months) until the best before date written on the packages.

**Table 1:** Protein, carbohydrate and fat composition of infant formulas according to the information provided by the manufacturer.

**Powder formulas**^**a**^ g per 100 ml ready to eat product						
	**Products**	**Other sources of protein**	**Recommended age from**	**Protein**	**Carbohydrate**	**Fat**
**1**	Manufacture A	Oat	6 months	2.2	8.6	2.5
**2**	Manufacturer A	Maize	6 months	1.7	9.4	2.4
**3**	Manufacturer B	Oat	6 months	1.8	8.5	3
**4**	Manufacturer B	Oat	1 year	2.2	8	2.5
**Liquid formulas**^**b**^ g per 100 ml liquid						
**5**	Manufacturer A	Oat	6 months	1.9	9	2.8
**6**	Manufacturer A	Maize	6 months	2.3	12	2.6
**7**	Manufacturer B	Oat	6 months	0.3	7.7	2.9
**8**	Manufacturer B	Oat	1 year	2.3	8.2	2.4
**9**	Manufacturer C	Oat	6 months	1.9	8.8	2.9
**10**	Manufacturer C	Oat	1 year	2.4	8.2	2.6

^a^ Powder infant formulas were vacuum packed after purchasing and placed into freezer -20°C until analysis.

^b^Liquid formulas were freeze-dried (Labconco, Missouri, USA), vacuum packed and put into freezer -20°C before further analysis.

### Methods

#### Determination of protein

Protein contents of the products were determined using a protein analyzer, Thermo Fisher Scientific Flash EA 1112 N series (Waltham, MA, USA), which operated according to the combustion technique, also known as the modified Dumas method. The instrument determined the nitrogen concentration and the relative protein content. The sample weight was 50 mg and the analysis was carried out in duplicate.

#### Determination of carbohydrates

Samples were analyzed for the determination of glucose, fructose, lactose and maltose. The analysis was carried out in duplicate using AOAC method 982.14.

#### Determination of water content

Water content (or the dry matter) of the samples were determined using the standard method of the International Dairy Federation [19]. Briefly, samples were dried in an oven (Termaks, Bergen, Norway) at 102°C for 2 hours. Following this, they were kept in a desiccator for one hour to reach the constant weight. 1 g of sample was utilized for each replicate and this analysis was carried out with two replicates.

#### Measurement of water activity (a_w_)

Water activity of the samples were measured using Aqua-lab water activity meter Series 3TE (Decagon Devices Inc., Washington, USA). 1 g of sample was used for each measurement and the analysis was performed with two replicates.

#### Determination of available lysine

The dye-binding method with Acid-orange 12 was employed for the determinations of available lysine is fully described and validated in our previous work [6] and is based on the work presented by Hurrell, Lerman & Carpenter, with slight modifications [20]. The method was successfully applied on a variety of skim milk powders in our previous work [21] and verified to be suitable for quantification of available lysine.

Briefly, 300 mg of sample were mixed with 2 ml sodium acetate solution for 20 min on an orbital lab shaker (type 3005, GFL, Gesellschaft für Labortechnik, Burgwedel, Germany). Then, 0.2 ml propionic anhydride were added to half of the flasks and the mixing was continued for another 20 min. Subsequently, 40 ml of the dye solution (with concentration of 1.36 mg/ml) were added and mixed for 1h. The analysis continued with the centrifugation step (5000 rpm, 10 min) by an Optima LE-80 k ultracentrifuge (Beckman Coulter, Bromma, Sweden) and the absorbance at 475 nm of the supernatants were measured using a Varian Cary 50 UV-Vis spectrophotometer (Agilent Technologies, CA, USA) and concentration of the available lysine was calculated using the equation from the calibration curve y = 47.37 x + 0.01 (*R*^2^ = 0.9999). The analysis was carried out with three replicates.

#### Determination of CML

The samples were prepared by hydrolyzing 0.3 g sample for 24 h at 110°C using 2 ml 6M HCl, together with an isotope labelled d4-CML (Larodan Fine Chemicals AB, Malmö Sweden) as the internal standard. To protect samples from oxidation during hydrolysis, the test tubes with samples in HCl were flushed with nitrogen (bubbling nitrogen gas through the sample) and sealed before incubation. Following hydrolysis, extraction of CML was performed using solid phase extraction (TelosneoPCX, Teknolab Sorbent AB, Västra Frölunda, Sweden). The SPE extracts were then evaporated to dryness, reconstituted in 200 μl 5mM NFPA in H2O and analyzed using LC-MS/MS [18].

The quantification of CML was performed using high-pressure liquid chromatography mass spectrometry (HPLC-MS/MS). Instruments used were Accela UHPLC pump with autoinjector coupled to LTQ VelosPro Orbitrap massspectrometer (Thermo Scientific, Waltham, USA). For data quantification and evaluation XcaliburTM 2.2 software (Thermo Scientific) was used. The MS/MS was run in positive electrospray ionization ion trap mode, detecting two Selected Reaction Monitoring (SRM) transitions for CML and two for the internal standard. Solid phase extraction, chromatographic parameters, ion source parameter and the SRM transitions are the same as described by Tareke et al., 2013.

The calibration curve was prepared with 0, 1, 2.5, 10, 50, 100 and 500 μg/ml CML and fixed amounts (100μl) of IS (d4-CML) with 100 μg/ml concentration (R^2^ = 0.9991). Samples containing higher concentrations of CML than the highest concentration in the calibration curve were diluted to ensure the analysis was carried out over the calibrated concentration range.

### Statistical analysis

Analysis of available lysine were conducted with three replicates, the determination of CML was performed using two replicates, and the results are reported as mean ± standard error. Student’s t-test (two samples assuming unequal variances) at 95% significance level was used to assess statistical significance of the data.

## Results and discussion

### Protein contents of infant formulas

Protein contents of the products measured using protein analyzer, are presented in Table 2.

**Table 2:** Protein content of the infant formulas determined using a protein analyzer. The Results are the Mean of Two Replicates ± Standard Error.

	Protein content % (n = 2) ± SE
**1**	10.78 ± 0.05
**2**	11.26 ± 0.05
**3**	12.09 ± 0.00
**4**	12.12 ± 0.18
**5**	12.52 ± 0.03
**6**	12.80 ± 0.16
**7**	12.40 ± 0.16
**8**	15.60 ± 0.07
**9**	13.07 ± 0.05
**10**	14.84 ± 0.43

### Composition of carbohydrates

In Table 3, results of sugar analysis are presented.

**Table 3:** Sugar content of the infant formulas. The Results are the Average of Two Replicates ± Standard Error.

	Glucose g/100 g (n = 2) ± 25%	Fructose g/100 g (n = 2) ± 25%	Lactose g/100 g (n = 2) ± 15%	Maltose g/100 g (n = 2) ± 15%
**1**	2.67	< 0.04	10.8	2.89
**2**	0.17	< 0.04	15.0	0.54
**3**	< 0.04	< 0.04	31.8	<0.04
**4**	0.33	< 0.04	24.2	0.65
**5**	0.31	< 0.04	12.7	5.85
**6**	0.33	< 0.04	15.1	1.19
**7**	0.05	< 0.04	31.5	0.87
**8**	0.05	< 0.04	29.5	0.94
**9**	0.26	< 0.04	25.9	13.1
**10**	0.31	< 0.04	20.0	14.0

Among the analyzed carbohydrates there is a large variation on the content of each sugar among samples (glucose: <0.04–2.67 g/100g, lactose: 10.8–31.8 g/100g, maltose: < 0.04–14.0 g/100g), independent of being a powder or liquid formulation, but seemingly more related with the manufacturer. Within the sugars analyzed it was not possible to find a relation with the content of CML. However, it does not disregard the possibility that free galactose could contribute to the formation of CML, but this was not possible to access with the method of analysis used and remains to be investigated.

### Water content and water activity

The infant formulas were also analyzed with respect to water content and water activity. These data were utilized in the further calculations regarding available lysine and CML contents (Table 4).

**Table 4:** Results of water content and water activity regarding the infant formulas.

	Powder formulas	Water content % (n = 2) ± SE	Water activity (a_w_) at 20 ºC
**1**	Manufacturer A	2.79 ± 0.07	0.308
**2**	Manufacturer A	3.14 ± 0.04	0.306
**3**	Manufacturer B	1.32 ± 0.03	0.232
**4**	Manufacturer B	2.12 ± 0.15	0.260
	**Liquid formulas**^**a**^		
**5**	Manufacturer A	0.07 ± 0.03	0.076
**6**	Manufacturer A	0.06 ± 0.00	0.078
**7**	Manufacturer B	0.22 ± 0.02	0.054
**8**	Manufacturer B	0.21 ± 0.08	0.045
**9**	Manufacturer C	1.71 ± 0.13	0.253
**10**	Manufacturer C	1.74 ± 0.01	0.251

^a^ It must be noted that liquid formulas were freeze-dried before being analyzed.

Water is a crucial factor in the progression of the Maillard reaction with its impact on glass transition temperature. Glass transition is the phase in which the lactose is transformed from a glassy and stable state to a rubbery state, where the reaction has the highest rate [22]. Furthermore, water may be produced with the progression of the Maillard reaction, and this leads to the increase in the water activity and water content of the product [23]. It was shown that the reaction has the highest rate at intermediate a_w_ 0.5–0.7 [24].

### Indication of early stage of Maillard reaction in available lysine

To evaluate whether the reaction is initiated, available lysine may provide reliable information. Traditionally, the Maillard reaction is divided into 3 steps. The interaction of lactose with lysine and the formation of the Amadori product is regarded as the *early* stage of the reaction. Degradation of the Amadori product and the formation of molecules such as CML, hydroxymethyl furfural (HMF), β-pyranone, 3-furanone, reductones, α-dicarbonyls, cyclopentenone, galactosylisomaltol and acetylpyrrole is considered as the *advanced* stage of the reaction and, finally, the generation of Melanoidins is the *final* stage [23]. In this study available lysine served as an indicator; to compare powder and liquid infant formulas and to assess the effect of different processing (spray-drying in case of powder and sterilization for the liquid formulas) on the commencement of the reaction. It has been reported that sterilization and spray-drying induce the Maillard reaction during the processing of infant formulas [10, 13]. Table 5 provides the results of available lysine, both presented based on dry matter and protein.

**Table 5:** Available lysine content of the infant formulas determined by a dye-binding method with three replicates.

Samples	Powder formulas	Available lysine % in dry matter (n = 3) ± SD	Available lysine % in protein (n = 3) ± SD
**1**	Manufacturer A	0.95 ± 0.06	8.13 ± 0.05
**2**	Manufacturer A	1.13 ± 0.06	9.75 ± 0.49
**3**	Manufacturer B	0.96 ± 0.02	7.74 ± 0.18
**4**	Manufacturer B	1.02 ± 0.02	8.28 ± 0.16
	**Liquid formulas**		
**5**	Manufacturer A	0.95 ± 0.02	7.55 ± 0.16
**6**	Manufacturer A	0.99 ± 0.01	7.76 ± 0.07
**7**	Manufacturer B	1.10 ± 0.02	8.86 ± 0.12
**8**	Manufacturer B	1.28 ± 0.04	8.16 ± 0.28
**9**	Manufacturer C	1.06 ± 0.02	7.97 ± 0.19
**10**	Manufacturer C	1.25 ± 0.03	8.30 ± 0.21

Since in our previously published works available lysine contents have been reported based on dry matter [6, 21], we use this unit for further discussion. Nevertheless, available lysine contents are also reported based on protein content to make it easy to compare with other studies. The conclusions can be drawn from these results is that powder and liquid infant formulas contain similar available lysine concentrations regardless of the manufacturer. Furthermore, considering that the concentrations of available lysine in these products are in the range 0.95–1.28% in dry matter, the infant formulas show 27.14–36.57% decrease in the available lysine, compared to the reference skim milk powder sample. The reference sample in this study was skim milk powder which was not exposed to any heat-treatment, the skim milk was collected after the fat separation step and freeze-dried. This sample contained 3.54% ± 0.09 available lysine based on dry matter.

Birlouez-Aragon et al. also showed that there was no significant difference between the liquid and powder infant formulas with respect to available lysine. They reported a decrease of 20% in the available lysine compared to raw cow’s milk [2]. Our results are slightly higher than the results reported by Contreras-Calderon. They showed that the average of available lysine in the infant formulas was 4.03–6.43% based on protein [25]. This can be explained by difference in the composition and heat processing applied on the products as well as the method that was employed for the determination of available lysine. In another study the available lysine loss as a result of processing in infant cereals ranged between 14–29% [9]. Similarly, Ferrer et al. reported a loss of 15.2–26.7% in the infant formulas, in comparison with the raw cow’s milk [10]. What can be understood from the present and also the previous studies is that infant formulas are more liable to the Maillard reaction and its consequences due to their composition, the presence of other provoking ingredients such as iron and vitamin C, and the application of rigorous heat treatments such as sterilization. In other words in order to mimic the composition of human milk (7.1% lactose, 40:60 casein to whey protein ratio) lactose and whey powder, both Maillard potential stimulants, are added to cow’s milk. Furthermore, to guarantee the microbiological safety of the products aimed for this sensitive group of population the heat applied in the form of in-bottle sterilization or spray-drying is more rigorous [1, 5]. The substantial effect of heat processing such as sterilization on the Maillard reaction has been well discussed in the literature [1, 10, 26]. Addition of vitamin C (one of the proposed pathways for formation of CML) and poly unsaturated fatty acids in the form of vegetable oils may lead to further progression of the Maillard reaction [26].

### Determination of CML

**Fig 1:**
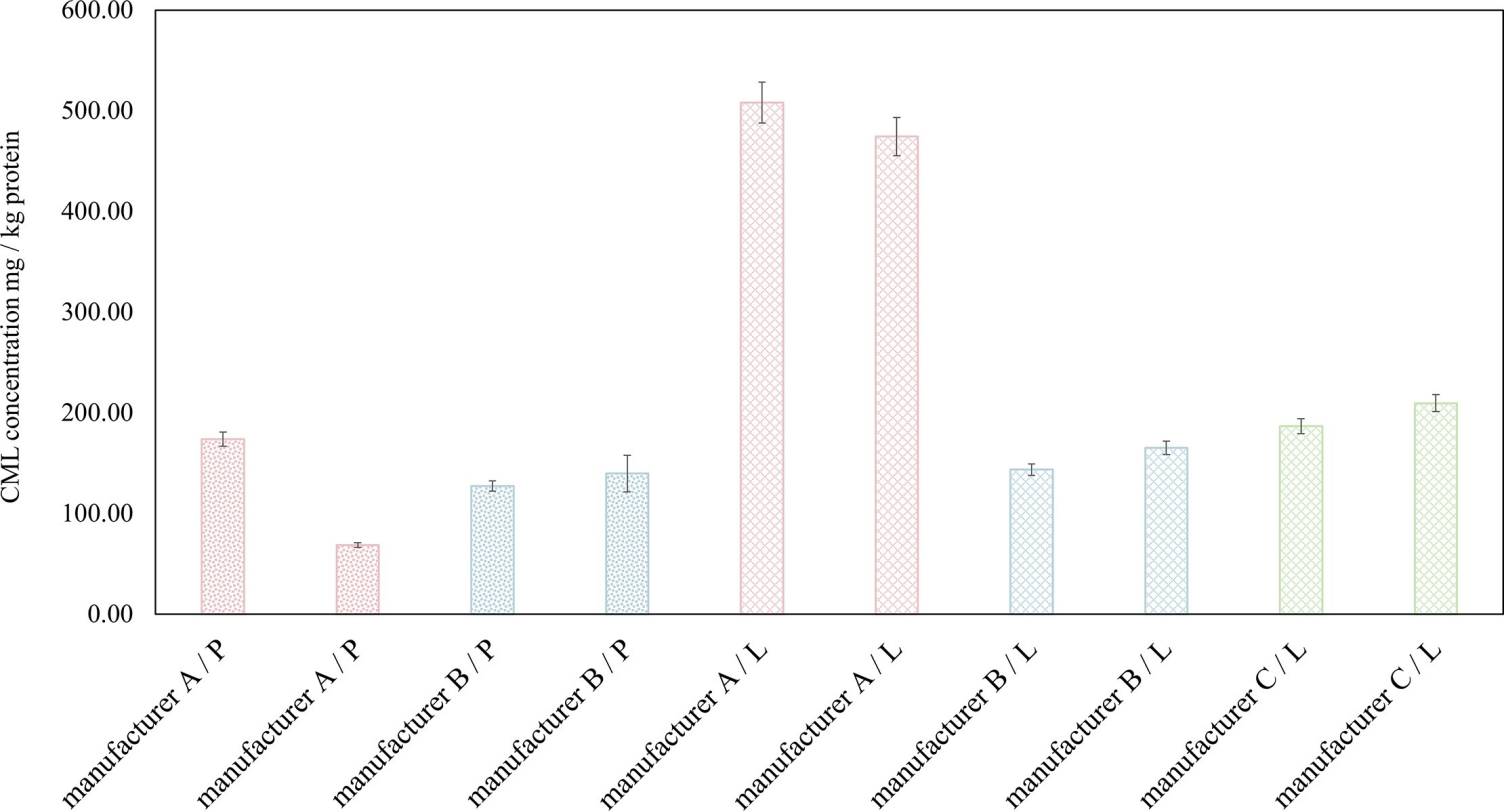
Studying the advanced stage of the reaction in the infant formulas using the indicator CML. CML Concentration is Calculated in mg / kg Protein (n = 2). The Error Bars Represent the Standard Errors. The Infant Formulas are from Three Manufacturers A, B and C. P Stands for Powder and L Means Liquid.

After studying the early phase of the reaction i.e., the study of the reaction precursors, it was logical to try to understand how the reaction status was in the advanced stage. The importance of this part of the study was not only to gain insight into the advanced phase of the Maillard reaction, but also the role of CML as an identified AGE molecule, as AGEs have been associated with risk factors for diabetes complications and other autoimmune diseases [5, 27]. CML was analyzed using the procedure established by Tareke et al. 2013 with two replicates. The analytical performance was assessed by considering the accuracy of the LC-MS/MS measurements using a standard sample with 10 μg/ml CML which was 115 ± 4%. The results of CML concentration in the 10 infant formulas are presented in Fig 1.

Unlike analysis of available lysine in these products (section 3.3), where the available lysine content of the infant formulas were quite similar, the CML concentration are in a broad range of 68.77–507.99 mg / kg protein. In the case of manufacturer A, the liquid infant formulas had significantly higher CML content compared to the powder products (p < 0.05). This may be explained by difference in handling/processing procedure applied on the products. This should be further investigated in the future.

Our results are in agreement with the previous studies. Hartkopf et al. reported that CML concentration was 50–200 mg/kg protein in the infant formulas [28]. In another study it was shown that 16 powder infant formulas contained 50–700 mg CML in kg protein. It was also concluded that CML concentration in the infant formulas were 28–389 times higher than the fresh human milk [29]. In another study by Dittrich et al. CML contents of 8 infant formulas available on the German market was reported to be 514–11372 ng / ml and 35 times higher than the CML concentration in the human milk. [14]. Similarly, Troise et al. reported the CML concentration of 80–140 mg /kg protein regarding commercial powder infant formulas [30]. As it was explained before, infant formulas show higher sensitivity towards the Maillard reaction, and this is mainly due to their composition which mimics the composition of human milk as well as the application of rigorous heat treatments. AGE content of these products may increase even more during storage, considering that these products mostly have prolonged shelf-life. It was shown in our previous study with skim milk powder that concentration of CML increased several times during storage at realistic storage conditions [31]. With respect to the spray-dried skim milk powder stored at 52% RH, 30°C after 30 days, the CML concentration reached approximately 3000 mg/kg protein and 24500 mg/kg protein after 200 days. This was, however, the accelerated storage condition. When the samples of skim milk powders were stored at 33% RH, 30°C, concentration of the CML after 30 and 200 days storage was around 400 and 2500 mg/kg protein respectively, indicating the importance of controlling every individual parameter such as temperature and RH throughout the storage. This is something that should be the priority of the future studies with respect to infant formulas.

## Concluding remarks

In summary, status of the Maillard reaction both the early and advanced phases of the reaction, were investigated in this study regarding commercial infant formulas available on the Swedish market. Ten powder and liquid infant formulas were analyzed with respect to available lysine, the indicator of early stage of the reaction, and CML as a well-characterized AGE molecule, a marker of the advanced phase of the Maillard reaction. The powder and liquid formulas contained similar available lysine contents in the range of 0.95–1.28% dry matter. Regarding CML, the concentrations were in a wide range of 68.77–507.99 mg/kg protein. In other words CML content of infant formulas available on the Swedish market can vary 7 fold, depending on the physical form and the manufacturers. Due to the higher liability of infant formulas to the Maillard reaction and sensitivity of the target group (infants), impact of the storage of these products at realistic storage conditions should be the priority of future studies.
